# Nutrient patterns in relation to insulin resistance and endothelial dysfunction in Iranian women

**DOI:** 10.1038/s41598-024-53263-1

**Published:** 2024-02-03

**Authors:** Farimah Dehghani, Zahra Hajhashemy, Ammar Hassanzadeh Keshteli, Ahmadreza Yazdannik, Ebrahim Falahi, Parvane Saneei, Ahmad Esmaillzadeh

**Affiliations:** 1 Department of Community Nutrition, School of Nutritional Sciences and Dietetics, Tehran University of Medical Sciences, P.O. Box 14155-6117, Tehran, Iran; 2 Department of Community Nutrition, School of Nutrition and Food Science, Nutrition and Food Security Research Center, Isfahan University of Medical Sciences, PO Box 81745-151, Isfahan, Iran; 3 Students’ Research Committee, Isfahan University of Medical Sciences, Isfahan, Iran; 4 Department of Medicine, University of Alberta, Edmonton, Canada; 5 Department of Critical Care Nursing, School of Nursing and Midwifery, Isfahan University of Medical Sciences, Isfahan, Iran; 6 Department of Nutrition, School of Health and Nutrition, Lorestan University of Medical Sciences, Khorramabad, Iran; 7 Obesity and Eating Habits Research Center, Endocrinology and Metabolism Molecular-Cellular Sciences Institute, Tehran University of Medical Sciences, Tehran, Iran; 8 Endocrinology and Metabolism Research Center, Endocrinology and Metabolism Clinical Sciences Institute, Tehran University of Medical Sciences, Tehran, Iran

**Keywords:** Disease prevention, Nutrition

## Abstract

Prior studies have mainly focused on the association of one specific nutrient with insulin resistance (IR) and endothelial dysfunction and limited studies have assessed the association with different nutrient patterns (NPs). We examined the association between various NPs and IR and endothelial dysfunction among Iranian women. This cross-sectional study was carried out on a sample of 368 female nurses. A 106-items food frequency questionnaire (FFQ) was applied for dietary assessments. Using factor analysis, the relationships between NPs and markers of insulin resistance (HOMA-IR, HOMA-β, and QUICKY), and endothelial dysfunction (E-selectin, sICAM-1, and sVCAM-1) were assessed. Mean age and body mass index of participants were respectively 35.21 years and 24.04 kg/m^2^. Three major NPs were identified. NP1, named as “dairy, fruits, and vegetables” had high values of potassium, folate, vitamins A and C, magnesium, and beta carotene. No significant association was observed between this NP and insulin resistance or endothelial dysfunction indices. The second NP was full of chromium, selenium, copper, vitamin B6, monounsaturated fatty acid (MUFA), thiamin, vitamin D, and iron. Adherence to NP2 (named “legumes, nuts, and protein foods”) was associated with lower values of insulin (6.8 ± 1.1 versus 8.4 ± 1.1, P = 0.01), homeostasis model assessment-Insulin resistance (HOMA-IR) (1.3 ± 0.2 versus 1.7 ± 0.2, P = 0.02), and vascular adhesion molecule 1 (VCAM-1) (444.2 ± 27.9 versus 475.8 ± 28.4, P = 0.03). However, adherence to the third NP, rich in saturated fatty acid (SFA), cholesterol, sodium, zinc, vitamin E, and B12, described as “animal fat and meat + vitamin E”, was associated with higher amounts of homeostasis model assessment-β (HOMA-β) (531.3 ± 176.2 versus 48.7 ± 179.8, P = 0.03). In conclusion, following the NP2, correlated with higher intakes of chromium, selenium, copper, vitamin B6, MUFA and thiamin was associated with lower values of insulin, HOMA-IR, and sVCAM-1. Adherence to NP3, rich in SFA, cholesterol, vitamin E, vitamin B12, and zinc was associated with higher levels of HOMA-β.

## Introduction

Endothelial dysfunction is characterized by a reduction in nitric oxide (NO) and a loss in endothelial cell properties^1^. This disorder is one of the main mechanisms of developing cardiovascular diseases^2^, certain cancers^3^, and metabolic syndrome (MetS)^4^. Moreover, the role of endothelial dysfunction in pathology of type 2 diabetes and insulin resistance (IR)^5^ has been investigated in several studies^6–8^. IR is described as an impaired response of skeletal muscles and liver to circulating insulin^9^. IR can be responsible in etiology of a variety of diseases from hepatic steatosis^10^ to thyroid disorders^11^ and Alzheimer’s diseases^12^.

An unhealthy lifestyle, consisting of smoking, insufficient physical activity^13^, and unhealthy dietary patterns are claimed to be the most prevalent risk factors for IR and endothelial dysfunction^14,15^. The universal characteristics of multiple nutrients have brought a new insight. For instance, it has been suggested that IR could be affected by several nutrients such as vitamin D, chromium^16^, magnesium^17^, fiber^18^, dietary fats such as polyunsaturated and omega 3 fatty acids^19,20^ and specific polyphenols such as anthocyanins^21^, resveratrol^22^, and quercetins^23,24^. Additionally, endothelial dysfunction could be influenced by nutrients such as magnesium^25^, flavanol^26^, vitamins C and E^27^, and lycopene^28^.

Prior studies have mainly focused on the association of one specific nutrient with outcomes such as IR, and endothelial dysfunction. Since nutrients usually are not consumed distinctly, evaluating the association between special combinations of different nutrients and outcomes of interest might provide a better insight. However, few studies have been carried out to evaluate the association between nutrient patterns and IR and endothelial function. For instance, a prospective cohort study has assessed the association between 5 nutrient patterns and risk of insulin-related disorders^24^. They illustrated that higher adherence to a nutrient pattern, rich in vitamins A, C, B6, potassium, and fructose had favorable effects on insulin, homeostasis model assessment-Insulin resistance (HOMA-IR), and Homeostatic Model Assessment of insulin Sensitivity (HOMA-S), during 3 years of follow-up. Thus, the present study aimed to estimate the association of nutrient patterns with endothelial function and IR in Iranian women.

## Methods

### Study design and participants

This cross-sectional study was conducted on a population of 368 female nurses working in 7 hospitals in Isfahan city. A multi-stage cluster random sampling method was used for selecting these participants. Serum insulin levels (with an SD of 6.54 among Iranians) were considered as the main dependent variable for estimating the total sample size^29^. Then, by considering type 1 error of 5%, and design effect of 1.25, a total number of 407.5 subjects were estimated to be required for this study. First, 510 females older than 30 years were randomly invited to participate in the study; 30 nurses rejected to take part in the study. So, 480 women agreed to participate in our study. We excluded 2 participants that did not complete over 70 items of dietary questionnaire. Moreover, 9 women with a total energy intake of less than 800 or over 4200 kcal/day, 26 women with a previous history of diabetes, cancer, stroke, and CVD, 16 women consuming medications that could change serum glucose values and 59 subjects with incomplete data were excluded from the study. Finally, this analysis was carried out on data from 368 female nurses. Each participant signed a written consent form. All methods of the current study were carried out according to the relevant guidelines and regulations. The present study’s approach has been approved by the ethics committee of the Tehran University of Medical Science (IR.TUMS.MEDICINE.REC.1400.178).

### Dietary assessment

A validated semi-quantitative dish-based FFQ was applied for assessment of common food intakes^30^. This FFQ included 106 food items and dishes and the participants were asked to report how often they have used these food items during the last year. Nine options ranging from “no or less than once in a month” to “more than 12 times in a day” were considered for each food item. A trained nutritionist instructed people on how to complete the FFQ. The validity and reliability of the FFQ were previously reported^30^. Additionally, the validity and reproducibility of the applied FFQ in the measurement of the average consumption of foods^31^, food groups^32^, and nutrients^33^ have been proven in the previous investigations. The US Department of Agriculture database (USDA) was used to calculate the total daily energy and nutrient intakes of each participant. Nutrient contents of some special foods were added to this software. The total energy and nutrient intake of each individual was computed by adding up energy intake and nutrients of all food items.

### Assessment of biomarkers

Fasting blood samples were collected for measurement of serum concentration of insulin, blood glucose, and adhesion molecules including E-selectin, soluble intercellular adhesion molecule (sICAM-1), and soluble vascular adhesion molecule 1 (sVCAM-1). These blood samples were centrifuged for 30–45 min after collection. Then, serums were kept at − 80 to be used for the analysis. We used available commercial kits by ELISA method (Biosource International and Bender Med Systems) for assessment of sICAM-1 (nearest to 0.6 mg/dL), sVCAM-1 (nearest to 2.3 mg/dL), and E-selectin (nearest to 0.3 mg/dL). We measured fasting blood glucose (FBG) through the use of an enzymatic calorimetric (a method that assesses FBG through glucose oxidase activity). Serum insulin was also estimated through the ELISA method (Bender Med System). Then, we assessed insulin resistance and insulin sensitivity, through the following formulas:

HOMA-IR = FBS (mmol/L) × Insulin (µmol/mL)/22.5^24^.

HOMA-β = (20 × insulin in mIU/mL)/ (FBG in mmol/L − 3.5).

QUICKY = 1/(log (fasting insulin (µU/mL) + log (fasting blood glucose (mg/dL))^34^.

### Assessment of other variables

Socioeconomic variables including the number of family members, educational level, residual status, number of bedrooms in their house, being a house owner, number and types of their cars, salary, and other sociodemographic properties such as age, marital status, menopause status, previous history of diseases, habits of taking medications or supplementations and smoking were assessed by using a self-administrated questionnaire. Body weight was measured by a digital scale (nearest to 0.1 kg), while subjects were shoeless and wearing light clothes. A tape measure was applied for evaluating standing status height. Then, body mass index (BMI) was calculated through the following formula: weight (in kilograms)/height (in meters) squared. The short form of the International Physical Activity Questionnaire (IPAQ)^35^ was used for estimating daily physical activity in MET-hour per week.

### Statistical analysis

Major nutrient patterns were extracted by performing factor analysis and entering 35 macro- and micro-nutrients in the analysis; these 35 nutrients were determined based on some previous publications in this regard^24,36,37^. Kaiser–Meyer–Olkin (KMO) test was applied to find out if the distribution of nutrients could be strong enough to use principal components. Factors with eigenvalues > 2 were considered as significant to extract major nutrient patterns. Scree plot was also used to identify the main nutrient patterns. Varimax rotation was conducted to extract independent nutrient patterns. Continuous and categorical characteristics of subjects were classified across tertiles of each nutrient pattern through the use of one-way ANOVA and chi-square tests, respectively. Mean dietary intakes of energy, food groups, and nutrients of participants across tertiles of nutrient patterns were obtained by ANCOVA. Mean values of glycemic factors and markers of insulin resistance and endothelial function across tertiles of nutrient patterns were estimated through ANCOVA in four models. This relationship was controlled for age and energy intake in the first model. Physical activity (MET-h/week), current corticosteroids and OCP intake (yes/no), marriage status (categorical), menopausal status (yes/no), systolic blood pressure (SBP), diastolic blood pressure (DBP), and socioeconomic status (categorical) were additionally controlled in the second model. Additional adjustment for BMI was conducted in the third model. In model 4 for association of nutrient patterns and glycemic factors and insulin resistance, additional adjustment was done for endothelial indices (E-selectin, sICAM-1, and sVCAM-1). While for association of nutrient patterns and endothelial markers, further adjustment was done for blood glucose and lipid profiles including serum triglyceride, serum total cholesterol, HDL-c, and LDL-c, in model 4. P values < 0.05 were assumed as statistically significant. Linear association between tertiles of nutrient patterns and indices of insulin resistance and endothelial function was assessed by linear regression analysis in both crude and adjusted models. Version 26 of SPSS was applied to perform all analysis.

### Ethical approval and consent to participate

All participants provided an informed written consent. The study protocol was approved by the local Ethics Committee of Isfahan University of Medical Sciences in 2022 (IR.TUMS.MEDICINE.REC.1400.178).

## Results

**Figure 1:**
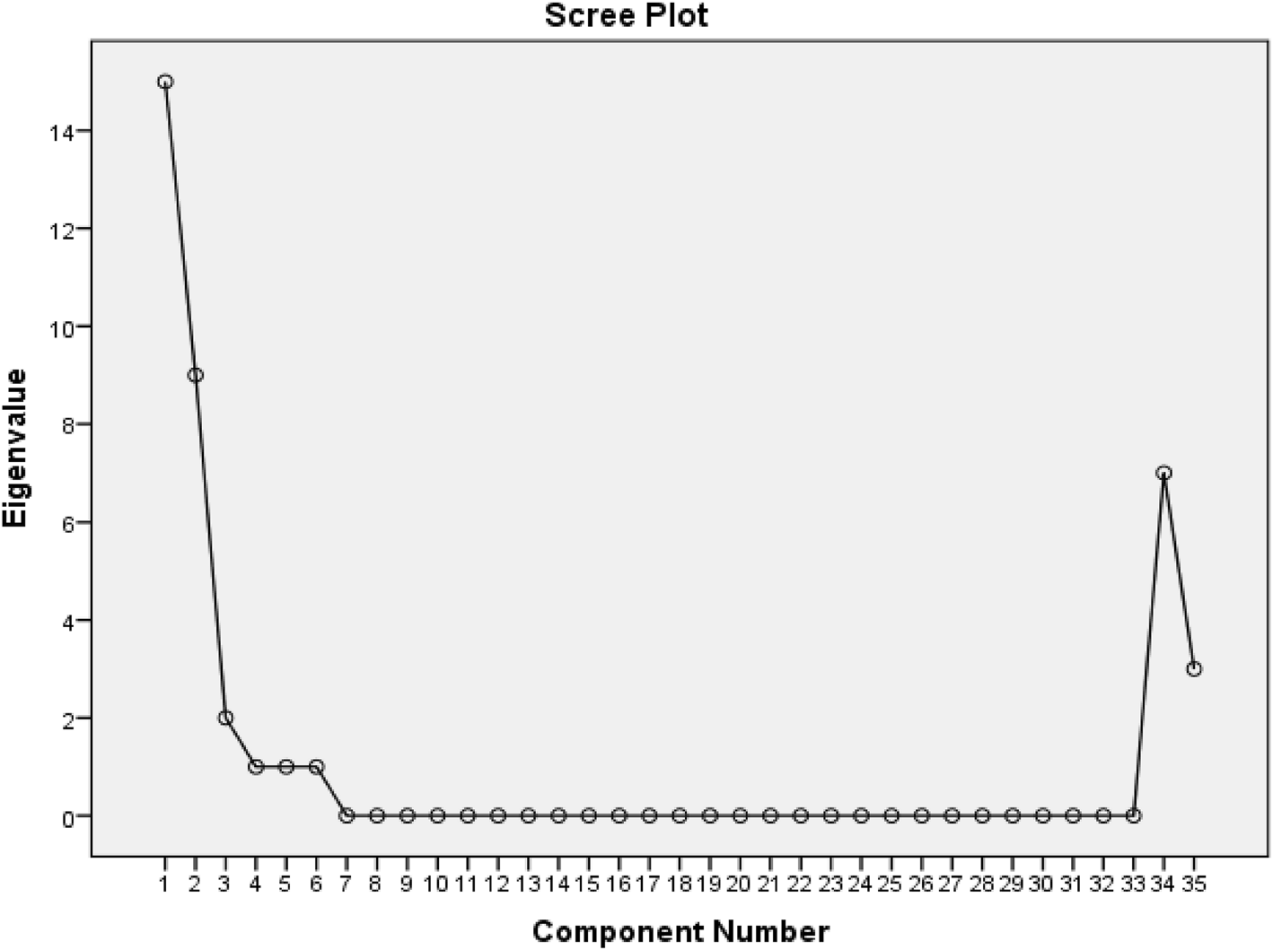
Scree plot for identifying major nutrient patterns in Iranian women.

The current study was conducted on 368 female nurses working in Iran hospitals. The mean age and BMI of participants were respectively 35.21 years and 24.04 kg/m^2^. Three nutrient dietary patterns have been extracted through factor analysis (Fig. 1). Factor loadings of each single nutrient in each nutrient pattern are provided in Table 1. Overall, 78.5% of all dietary changes have been explained through these three nutrient patterns. Nutrient pattern 1 was associated with greater amounts of potassium, folate, vitamin A, vitamin C, magnesium, beta carotene, pantothenic acid, sugar, phosphorus, riboflavin, biotin, vitamin K, calcium, and carbohydrate. This pattern has been supposed to be rich in dairy products, fruits, and vegetables. The second nutrient pattern was correlated with higher intakes of chromium, selenium, copper, vitamin B6, monounsaturated fatty acid (MUFA), thiamin, polyunsaturated fatty acid (PUFA), vitamin D, iron, and dietary fiber. This nutrient pattern was considered to be full of legumes, nuts, and protein foods. The third nutrient pattern was related to higher values of saturated fatty acid (SFA), cholesterol, vitamin E, sodium, vitamin B12, zinc, and protein. Therefore, this NP seemed to be correlated with higher consumption of animal fat and meat + vitamin E.

**Table 1:** Factor loadings and explained variances for major nutrient patterns (NPs).

	NP1 (dairy, fruits, vegetables)	NP2 (legumes, nuts, protein)	NP3 (animal fat and meat + vitamin E)
Potassium	0.935		0.226
Folate	0.931		0.216
Vitamin A	0.905		
Vitamin C	0.897		
Magnesium	0.888		0.363
Beta carotene	0.851		
Pantothenic acid	0.848		0.438
Sugar	0.806		
Phosphorus	0.786		0.480
Riboflavin	0.776		0.423
Biotin	0.772		0.446
Vitamin K	0.749		0.397
Calcium	0.740		0.340
Carbohydrate	0.698		0.267
Manganese	0.629	0.617	
Niacin	0.551		0.507
Chromium		0.989	
Selenium		0.989	
Copper		0.986	
Vitamin B6		0.982	
MUFA		0.977	
Thiamin		0.975	
Vitamin D		0.966	
Iron		0.947	0.239
PUFA		0.835	0.467
Dietary fiber	0.600	0.755	
Fluoride			
SFA		0.480	0.783
Cholesterol	0.303		0.719
Vitamin E	0.225		0.715
Sodium	0.312		0.706
Vitamin B12	0.248	0.386	0.704
Zinc		0.521	0.687
Protein		0.462	0.642
Molybdenum	0.484		0.531
Variance explained (%)	32.475	29.230	16.768
Cumulative explained variance (%)	32.475	61.705	78.473

Factor loadings < │0.20│ are not shown for simplicity. The Kaiser–Meyer–Olkin value was 0.85. Retained factors with Eigenvalues ≥ 2 were extracted as major NPs.

General features of the study subjects across tertiles of nutrient patterns are shown in Table 2. There was no significant difference in socio-demographic characteristics across tertiles of nutrient patterns 1 and 2. However, a marginally lower BMI (23.4 vs. 24.4, P = 0.05) and waist circumferences (79.1 vs. 82.1, P = 0.05) have been observed among subjects in the highest tertiles in comparison to those in the lowest tertile of NP3. Participants with menopause status were lower in the highest tertile compared to the lowest tertile of NP3 (2.2% vs. 10.7%, P = 0.01). Other socio-demographic characteristics were not significantly different between tertiles of NP3.

**Table 2:** General characteristics of study population across categories of nutrient pattern scores.

	Tertiles of NP1 score				Tertiles of NP2 score				Tertiles of NP3 score			
	T1 (n = 127)	T2 (n = 119)	T3 (n = 122)	P^a^	T1 (n = 117)	T2 (n = 123)	T3 (n = 128)	P^a^	T1 (n = 121)	T2 (n = 121)	T3 (n = 126)	P^a^
Age (years)	34.4 ± 7.1	35.5 ± 7.3	35.6 ± 7.2	0.36	34.5 ± 6.5	35.2 ± 7.1	35.8 ± 7.9	0.31	36.1 ± 7.4	35.2 ± 7.1	34.2 ± 6.9	0.08
Weight (kg)	61.6 ± 9.8	69.8 ± 81.0	63.4 ± 9.76	0.34	69.3 ± 81.8	63.1 ± 9.9	62.7 ± 9.1	0.45	63.9 ± 10.2	62.3 ± 8.8	68.8 ± 81.5	0.52
BMI (kg/m^2^)	23.8 ± 3.7	24.0 ± 3.2	24.2 ± 3.7	0.64	23.8 ± 3.6	24.1 ± 3.5	24.1 ± 3.5	0.65	24.4 ± 3.5	24.2 ± 3.4	23.4 ± 3.6	0.05
Waist circumference (cm)	80.4 ± 10.1	80.9 ± 9.9	81.3 ± 10.5	0.76	80.5 ± 9.7	81.7 ± 10.6	80.4 ± 10.1	0.52	82.1 ± 10.3	81.4 ± 9.8	79.1 ± 10.2	0.05
Systolic blood pressure (cmHg)	10.7 ± 1.06	10.8 ± 1.3	10.9 ± 1.1	0.51	10.8 ± 1.1	10.8 ± 1.07	10.9 ± 1.2	0.76	10.9 ± 1.2	10.9 ± 1.09	10.7 ± 1.01	0.68
Diastolic blood pressure (cmHg)	7.06 ± 0.87	6.9 ± 1.06	7.07 ± 0.97	0.42	6.9 ± 0.96	7.0 ± 0.84	7.09 ± 1.1	0.59	7.03 ± 1.09	7.06 ± 0.89	6.9 ± 0.94	0.78
Physical activity (MET-h/w k)	82.7 ± 90.2	76.8 ± 88.2	75.2 ± 81.1	0.86	83.7 ± 97.8	89.4 ± 94.04	60.9 ± 60.5	0.09	76.2 ± 74.7	82.03 ± 94.7	75.03 ± 87.4	0.86
Current OCP use [n (%)]	8 (5.8)	8 (5.8)	11 (8)	0.71	8 (5.8)	12 (8.7)	7 (5.1)	0.44	6 (4.4)	11 (8)	10 (7.3)	0.44
Current corticosteroid use [n (%)]	4 (2.9)	0 (0)	2 (1.5)	0.13	2 (1.5)	1 (0.7)	3 (2.2)	0.60	2 (1.5)	1 (0.7)	3 (2.2)	0.58
Menopausal [n (%)]	7 (5.1)	6 (4.3)	11 (7.9)	0.40	4 (2.9)	7 (5)	13 (9.4)	0.06	15 (10.7)	6 (4.3)	3 (2.2)	0.01
High socioeconomic status^b^ [n (%)]	26 (26.5)	24 (25.5)	31 (32.3)	0.15	27 (27.3)	26 (25.7)	28 (31.8)	0.90	30 (30.9)	24 (25.0)	27 (28.4)	0.62
Married [n (%)]	106(76.3)	99 (70.7)	100 (73)	0.57	105(75.5)	104 (74.3)	96 (70.1)	0.56	100(71.9)	102 (73.9)	103 (74.1)	0.90
People with overweight or obesity^c^ [n (%)]	54 (40.9)	45 (33.3)	56 (42.7)	0.24	50 (37.9)	50 (38.2)	55 (40.7)	0.87	58 (43.9)	55 (41.4)	42 (31.6)	0.09

Data are means ± SD or number (%).*Q* quartile, *BMI* body mass index, *MET-h/wk* metabolic equivalent-hour per week, *OCP* the oral contraceptive pill.

^a^Obtained from analysis of variance (ANOVA) for continuous variables and chi-square for categorical variables.

^b^High socioeconomic status was defined based on educational level, income, family size, being house owners, house area, number and kind of the car (s), number of bedrooms, and determination of who was in charge for the family.

^c^Defined as BMI ≥ 25 kg/ m^2^.

Usual dietary intakes of individuals across tertile of NPs are presented in Table 3. Consumption of total energy intake (P = 0.001), vegetables (P < 0.001), fruits (P < 0.001), low-fat dairy (P < 0.001), legumes and nuts (P < 0.001) and total dietary fiber (P < 0.001) were significantly higher among subjects in the highest vs. lowest tertile of NP1. Lower intakes of refined grains (P ˂ 0.001), oils (P = 0.002), protein (P ˂ 0.001), total fat (P = 0.001), SFA (P ˂ 0.001), MUFA (P = 0.01) and PUFA (P  ˂ 0.001) have also been observed in tertile 3 in comparison to tertile 1 of NP1. Participants in the highest tertile compared with the lowest tertile of the second nutrient pattern had higher intakes of energy (P = 0.01), vegetables (P  ˂ 0.001), fruits (P  ˂ 0.001), SFA (P = 0.03), protein (P  ˂ 0.001), carbohydrate (P  ˂ 0.001) and total dietary fiber (P  ˂ 0.001), and lower intakes of white meat (P = 0.04), refined grains (P = 0.04), cholesterol (P  ˂ 0.001) and sodium (P  ˂ 0.001). The third vs. first tertile of nutrient pattern 3 was associated with higher consumptions of energy intake (P = 0.001), white meat (P = 0.001), refined grains (P = 0.03), oils (P  ˂ 0.001), protein (P  ˂ 0.001), fats (P  ˂ 0.001), SFA (P  ˂ 0.001), cholesterol (P  ˂ 0.001) and sodium (P  ˂ 0.001), and lower intakes of vegetables (P  ˂ 0.001), fruits (P  ˂ 0.001), carbohydrate (P  ˂ 0.001) and dietary fiber (P  ˂ 0.001).

**Table 3:** Dietary intakes of study participants across tertiles of nutrient patterns.

	Tertiles of NP1 score				Tertiles of NP2 score				Tertiles of NP3 score			
	T1 (n = 127)	T2 (n = 119)	T3 (n = 121)	P^a^	T1 (n = 117)	T2 (n = 123)	T3 (n = 128)	P^a^	T1 (n = 121)	T2 (n = 121)	T3 (n = 126)	P^a^
Energy (Kcal/day)	2061.2 ± 64.8	2699.7 ± 64	3517.6 ± 64	0.001	2752.7 ± 82.1	2598.7 ± 81	2942.2 ± 81.1	0.01	2113.2 ± 66.6	2722.2 ± 67.1	3473 ± 67.3	0.001
Food groups												
Vegetables (g/day)	204.8 ± 19.0	314.2 ± 16.5	517.7 ± 19.1	˂ 0.001	248.6 ± 17.4	330.1 ± 17.2	457.8 ± 17.2	˂ .001	464.5 ± 19.5	331.7 ± 17.6	240.9 ± 20.01	˂ 0.001
Fruits (g/day)	209.6 ± 26.5	359.2 ± 23.0	601.3 ± 26.7	˂ 0.001	240.4 ± 23.3	379.0 ± 23.2	550.0 ± 23.1	˂ 0.001	576.2 ± 25.8	377.7 ± 23.4	215.9 ± 26.5	˂ 0.001
White meat (g/day)	85.9 ± 5.9	89.2 ± 5.1	93.5 ± 5.9	0.72	98.0 ± 5.2	91.1 ± 5.1	79.7 ± 5.1	0.04	71.8 ± 5.6	95.4 ± 5.1	101.7 ± 5.8	0.001
Red meats (g/day)	141.3 ± 8.4	131.5 ± 7.2	146.7 ± 8.4	0.35	147.0 ± 7.3	145.9 ± 7.3	126.8 ± 7.3	0.09	132.9 ± 8.09	151.4 ± 7.3	135.1 ± 8.3	0.15
Low-fat dairy (g/day)	222.1 ± 26.3	337.1 ± 22.8	540.0 ± 26.5	˂ 0.001	369.5 ± 24.8	379.0 ± 24.6	353.8 ± 24.6	0.77	370.8 ± 27.2	363.7 ± 24.6	367.6 ± 28.00	0.98
High-fat dairy (g/day)	49.0 ± 5.5	60.1 ± 4.7	71.9 ± 5.5	0.32	52.5 ± 4.8	63.5 ± 4.8	65.1 ± 4.8	0.13	61.4 ± 5.3	60.2 ± 4.8	59.7 ± 5.5	0.97
Refined grains (g/day)	445.1 ± 17.5	413.4 ± 15.2	309.3 ± 17.7	˂ 0.001	379.8 ± 15.8	420.0 ± 15.7	366.8 ± 15.7	0.04	353.6 ± 17.4	412.6 ± 15.7	401.1 ± 17.8	0.03
Whole grains (g/day)	66.6 ± 9.5	59.5 ± 8.2	60.0 ± 9.6	0.84	57.7 ± 8.4	65.7 ± 8.3	62.5 ± 8.3	0.79	74.1 ± 9.1	67.0 ± 8.2	44.6 ± 9.4	0.10
Legumes and nuts (g/day)	48.3 ± 3.4	57.8 ± 3.0	70.6 ± 3.4	˂ 0.001	56.5 ± 3.1	60.5 ± 3.0	59.9 ± 3.0	0.60	58.1 ± 3.4	59.7 ± 3.0	59.3 ± 3.4	0.93
Oils (g/day)	76.7 ± 2.8	73.2 ± 2.4	61.6 ± 2.8	0.002	73.4 ± 2.4	72.6 ± 2.4	65.4 ± 2.4	0.04	60.1 ± 2.6	71.5 ± 2.4	80.0 ± 2.7	˂ 0.001
Nutrients												
Protein (g/day)	171.8 ± 10.3	105.6 ± 8.98	78.9 ± 10.4	˂ 0.001	90.8 ± 9.1	104.4 ± 9.0	158.9 ± 9.0	˂ 0.001	57.7 ± 9.2	96.4 ± 8.3	202.4 ± 9.4	˂ 0.001
Carbohydrate (g/day)	314.4 ± 6.2	332.8 ± 5.4	343.1 ± 6.2	0.12	296.1 ± 5.0	335.3 ± 5.0	358.3 ± 5.0	˂ 0.001	378.5 ± 5.2	332.1 ± 4.7	279.0 ± 5.3	˂ 0.001
Fats (g/day)	123.3 ± 5.2	105.8 ± 4.5	92.0 ± 5.3	0.001	111.1 ± 4.7	103.6 ± 4.6	106.2 ± 4.6	0.51	91.7 ± 5.0	104.3 ± 4.5	125.1 ± 5.2	˂ 0.001
Saturated fatty acids (g/day)	30.4 ± 1.04	24.4 ± 0.90	21.5 ± 1.04	˂ 0.001	24.4 ± 0.9	24.3 ± 0.9	27.3 ± 0.9	0.03	17.7 ± 0.8	23.8 ± 0.8	34.8 ± 0.9	˂ 0.001
MUFA (g/day)	57.6 ± 8.4	33.6 ± 7.3	19.5 ± 8.4	0.01	34.1 ± 7.4	32.5 ± 7.4	43.5 ± 7.4	0.53	29.7 ± 8.1	32.6 ± 7.4	48.2 ± 8.3	0.28
PUFA (g/day)	52.3 ± 2.6	41.2 ± 2.2	32.6 ± 2.6	˂ 0.001	42.0 ± 2.3	39.8 ± 2.3	44.0 ± 2.3	0.45	34.5 ± 2.5	39.7 ± 2.3	51.9 ± 2.6	˂ 0.001
Cholesterol (mg/day)	252.6 ± 9.1	240.5 ± 7.9	228.0 ± 9.2	0.23	290.3 ± 7.4	223.5 ± 7.4	208.4 ± 7.4	˂ 0.001	174.8 ± 7.7	234.9 ± 6.9	312.6 ± 7.9	˂ 0.001
Sodium (mg/day)	4197.6 ± 127.7	3961.3 ± 110.7	3774.8 ± 128.4	0.10	4506.7 ± 107.8	3856.5 ± 107.0	3580.2 ± 106.9	˂ 0.001	3151.5 ± 112.1	3989.5 ± 101.4	4806.3 ± 115.0	˂ 0.001
Total dietary fiber (g/day)	18.6 ± 1.1	20.2 ± 1.0	26.0 ± 1.1	˂ 0.001	16.8 ± 0.9	20.6 ± 0.9	27.3 ± 0.9	˂ 0.001	26.7 ± 1.0	20.7 ± 0.9	17.4 ± 1.1	˂ 0.001

^a^Dietary intakes of foods and nutrients are reported. All values are means ± SE. *Q* quartile, *MUFA* monounsaturated fatty acid, *PUFA* polyunsaturated fatty acid.

^b^Obtained from analysis of variance (ANOVA).

Multivariable-adjusted mean ± SE of glycemic indices and insulin resistance markers across tertiles of nutrient patterns are reported in Table 4. The indices of glycemic profile and insulin resistance were not significantly different across tertiles of NP1. Subjects in the highest tertile of NP2 had significantly lower insulin levels (6.8 ± 1.1 vs. 8.4 ± 1.1, P = 0.006) in comparison to the lowest tertile in fully-adjusted model. Participants in the top tertile of NP2 compared with the bottom tertile had lower levels of HOMA-IR (1.3 ± 0.2 vs. 1.7 ± 0.2, P = 0.02), in the fully-adjusted model. Other glycemic indices were not significantly different across tertiles of NP2. Subjects in the highest tertile of NP3 in comparison to the lowest tertile, had higher levels of HOMA-β (542.0 ± 176.0 vs. 44.1 ± 175.0, P = 0.03), in the second model. This association was significant even after adjustment for all potential covariates (531.3 ± 176.2 vs. 48.7 ± 179.8, P = 0.03).

**Table 4:** Multivariable-adjusted glycemic profile and insulin resistance across tertiles of nutrient pattern scores.

	Tertiles of NP1 score				Tertiles of NP2 score				Tertiles of NP3 score			
	T1 (n = 127)	T2 (n = 119)	T3 (n = 122)	P	T1 (n = 117)	T2 (n = 123)	T3 (n = 128)	P	T1 (n = 121)	T2 (n = 121)	T3 (n = 126)	P
FBG (mg/dL)												
Crude	82.6 ± 1.1	81.9 ± 1.2	83.0 ± 1.1	0.78	81.2 ± 0.9	82.5 ± 1.3	83.7 ± 1.1	0.32	83.0 ± 1.4	82.1 ± 0.9	82.4 ± 0.9	0.85
Model 1^a^	82.4 ± 1.3	82.1 ± 1.2	82.7 ± 1.4	0.95	80.8 ± 1.2	82.5 ± 1.2	83.8 ± 1.2	0.23	83.7 ± 1.3	81.7 ± 1.2	81.8 ± 1.3	0.52
Model 2^b^	83.0 ± 2.7	81.1 ± 2.3	82.9 ± 2.9	0.82	81.1 ± 2.4	84.7 ± 2.4	81.1 ± 2.4	0.49	81.6 ± 2.7	82.1 ± 2.3	83.3 ± 2.7	0.91
Model 3^c^	82.7 ± 2.7	81.0 ± 2.4	83.2 ± 2.9	0.80	80.7 ± 2.4	84.9 ± 2.4	81.2 ± 2.4	0.46	81.3 ± 2.7	82.1 ± 2.4	83.4 ± 2.7	0.88
Model 4^d^	82.5 ± 2.8	80.5 ± 2.5	83.4 ± 2.9	0.73	80.5 ± 2.5	85.7 ± 2.5	80.2 ± 2.5	0.26	80.9 ± 2.8	81.9 ± 2.4	83.5 ± 2.7	0.82
Insulin (mU/L)												
Crude	9.7 ± 1.1	7.4 ± 0.5	8.4 ± 0.6	0.14	8.8 ± 1.2	9.0 ± 0.6	7.8 ± 0.4	0.56	9.1 ± 1.1	8.5 ± 0.6	7.9 ± 0.5	0.60
Model 1^a^	9.3 ± 0.9	7.2 ± 0.8	9.2 ± 0.9	0.15	8.9 ± 0.8	8.9 ± 0.8	7.9 ± 0.8	0.62	8.3 ± 0.9	8.4 ± 0.8	8.9 ± 0.9	0.90
Model 2^b^	10.6 ± 1.2	8.5 ± 1.1	7.1 ± 1.3	0.23	8.6 ± 1.1	10.7 ± 1.1	7.05 ± 1.1	0.08	7.5 ± 1.2	7.8 ± 1.0	11.0 ± 1.2	0.11
Model 3^c^	10.4 ± 1.2	8.4 ± 6.2	7.2 ± 1.3	0.28	8.4 ± 1.1	10.6 ± 1.1	7.0 ± 1.1	0.09	7.2 ± 1.2	7.8 ± 1.0	11.0 ± 1.2	0.10
Model 4^d^	10.4 ± 1.2	8.3 ± 1.1	7.3 ± 1.3	0.29	8.4 ± 1.1	10.8 ± 1.1	6.8 ± 1.1	0.06	7.2 ± 1.2	8.0 ± 1.1	10.7 ± 1.2	0.17
HOMA-IR												
Crude	2.0 ± 0.2	1.5 ± 0.1	1.7 ± 0.1	0.19	1.8 ± 0.2	1.8 ± 0.1	1.6 ± 0.1	0.63	1.9 ± 0.2	1.7 ± 0.1	1.6 ± 0.1	0.57
Model 1^a^	1.9 ± 0.2	1.5 ± 0.1	1.9 ± 0.2	0.16	1.8 ± 0.1	1.8 ± 0.1	1.6 ± 0.1	0.66	1.7 ± 0.2	1.7 ± 0.1	1.8 ± 0.2	0.92
Model 2^b^	2.2 ± 0.2	1.7 ± 0.2	1.5 ± 0.2	0.25	1.8 ± 0.2	2.2 ± 0.2	1.3 ± 0.2	0.04	1.5 ± 0.2	1.6 ± 0.2	2.3 ± 0.2	0.12
Model 3^c^	2.1 ± 0.2	1.7 ± 0.2	1.5 ± 0.3	0.32	1.7 ± 0.2	2.2 ± 0.2	1.3 ± 0.2	0.04	1.4 ± 0.2	1.6 ± 0.2	2.3 ± 0.2	0.10
Model 4^d^	2.1 ± 0.2	1.6 ± 0.2	1.5 ± 0.3	0.33	1.7 ± 0.2	2.3 ± 0.2	1.3 ± 0.2	0.02	1.4 ± 0.2	1.6 ± 0.2	2.2 ± 0.2	0.16
HOMA-β												
Crude	225.7 ± 65.5	129.7 ± 29.7	60.2 ± 76.6	0.15	228.8 ± 64.7	95.6 ± 77.7	101.8 ± 32.8	0.22	146.4 ± 37.6	66.7 ± 77.4	202.5 ± 60.4	0.28
Model 1^a^	232.6 ± 69.7	119.6 ± 63.3	51.5 ± 73.4	0.25	234.0 ± 65.3	78.5 ± 63.0	102.9 ± 61.8	0.18	85.6 ± 70.6	54.5 ± 64.1	259.4 ± 69.8	0.09
Model 2^b^	388.1 ± 181.7	181.9 ± 158.5	− 96.6 ± 192.8	0.27	288.3 ± 162.3	− 68.8 ± 161.5	261.1 ± 162.5	0.24	44.1 ± 175.7	− 101.7 ± 154.9	542.0 ± 176	0.03
Model 3^c^	387.2 ± 184.5	162.3 ± 159.6	− 71.4 ± 194.5	0.32	254.7 ± 165.9	− 49.6 ± 165.1	267.0 ± 163.3	0.32	57.4 ± 178.5	− 116.2 ± 155.4	540.0 ± 176.3	0.02
Model 4^d^	407.2 ± 183.3	144.9 ± 162.6	− 72.3 ± 192.8	0.27	254.4 ± 167.8	10.3 ± 167.8	213.9 ± 166.5	0.56	48.7 ± 179.8	− 107.8 ± 157.5	531.3 ± 176.2	0.03
QUICKY												
Crude	0.37 ± 0.005	0.37 ± 0.004	0.37 ± 0.004	0.59	0.38 ± 0.005	0.37 ± 0.004	0.37 ± 0.004	0.22	0.37 ± 0.005	0.37 ± 0.004	0.37 ± 0.003	0.73
Model 1^a^	0.37 ± 0.005	0.37 ± 0.005	0.37 ± 0.006	0.63	0.38 ± 0.005	0.37 ± 0.005	0.37 ± 0.005	0.21	0.37 ± 0.005	0.37 ± 0.005	0.37 ± 0.005	0.73
Model 2^b^	0.36 ± 0.01	0.37 ± 0.009	0.38 ± 0.01	0.54	0.38 ± 0.009	0.36 ± 0.009	0.37 ± 0.009	0.30	0.38 ± 0.01	0.38 ± 0.009	0.36 ± 0.01	0.21
Model 3^c^	0.37 ± 0.01	0.37 ± 0.009	0.38 ± 0.01	0.62	0.38 ± 0.009	0.36 ± 0.009	0.37 ± 0.009	0.24	0.38 ± 0.01	0.38 ± 0.009	0.36 ± 0.01	0.20
Model 4^d^	0.37 ± 0.01	0.37 ± 0.009	0.37 ± 0.01	0.69	0.38 ± 0.009	0.36 ± 0.009	0.38 ± 0.009	0.09	0.38 ± 0.01	0.38 ± 0.009	0.36 ± 0.01	0.19

All values are means ± SE. P were obtained from analysis of covariance (ANCOVA). *Q* quartile, *FBG* fasting blood glucose, *HOMA-IR* homeostatic model assessment of insulin resistance, *HOMA-β* homeostatic model assessment of beta-cell function, *QUICKI* quantitative insulin sensitivity check index.

^a^Model 1: Adjusted for age and energy intake.

^b^Model 2: Further adjusted for physical activity (MET-h/wk), current corticoid steroids use (yes or no), current OCP use (yes or no), marital status (categorical), menopausal status (yes or no), systolic blood pressure, diastolic blood pressure, and socioeconomic status (categorical).

^c^Model 3: Further adjusted for BMI.

^d^Model 4: Additionally adjusted for markers of endothelial function (E-selectin, sICAM-1, and sVCAM-1).

Table 5 shows the multivariable-adjusted mean ± SE of endothelial function markers across tertiles of nutrient patterns. Individuals in the highest tertile in comparison to those in the lowest tertile of NP1 had higher levels of sICAM-1 in the crude model (223.7 ± 8.5 vs. 201.1 ± 6.4, P = 0.03). This significant difference disappeared after adjustment for all covariates in model 4. In the crude model, levels of E-selectin were lower in the highest tertile compared with the lowest tertile of NP2 (79.6 ± 3.1 vs. 98.6 ± 7.8, P = 0.01). However, there was no significant difference in E-selectin levels across tertiles of NP2, after controlling for potential covariates (84.9 ± 6.4 vs. 82.0 ± 6.3, P = 0.94). Individuals in the highest tertile of NP2 had also lower levels of sVCAM-1 in comparison to the lowest tertile, after adjusting for all potential variables (444.2 ± 27.9 vs. 475.8 ± 28.4, P = 0.03). Indices of endothelial function were not significantly different across tertiles of NP3, in both crude and fully-adjusted model.

**Table 5:** Multivariable-adjusted association between markers of endothelial function and tertiles of nutrient pattern scores.

	Tertiles of NP1 score				Tertiles of NP2 score				Tertiles of NP3 score			
	T1 (n = 127)	T2 (n = 119)	T3 (n = 122)	P	T1 (n = 117)	T2 (n = 123)	T3 (n = 128)	P	T1 (n = 121)	T2 (n = 121)	T3 (n = 126)	P
E-selectin (mg/L)												
Crude	94.5 ± 7.2	81.3 ± 3.0	82.4 ± 3.4	0.11	98.6 ± 7.8	81.4 ± 3.0	79.6 ± 3.1	0.02	84.2 ± 3.5	89.3 ± 7.6	85.3 ± 2.9	0.75
Model 1^a^	91.2 ± 5.5	81.6 ± 5.0	84.6 ± 5.8	0.42	95.8 ± 5.1	81.7 ± 5.0	81.0 ± 4.8	0.06	82.4 ± 5.6	86.7 ± 5.1	88.6 ± 5.6	0.76
Model 2^b^	87.8 ± 6.7	83.0 ± 6.0	79.2 ± 7.2	0.73	82.9 ± 6.1	84.0 ± 5.9	83.1 ± 6.2	0.99	80.0 ± 6.6	78.6 ± 5.9	91.6 ± 6.7	0.35
Model 3^c^	86.7 ± 6.8	83.2 ± 6.0	79.2 ± 7.3	0.80	82.9 ± 6.3	83.2 ± 6.0	83.0 ± 6.2	0.99	78.7 ± 6.7	78.8 ± 5.9	91.6 ± 6.7	0.34
Model 4^d^	85.2 ± 6.9	84.7 ± 6.1	79.2 ± 7.4	0.84	84.9 ± 6.4	82.3 ± 6.1	82.0 ± 6.3	0.94	79.4 ± 6.8	79.0 ± 6.0	90.7 ± 6.8	0.42
sICAM-1 (mg/L)												
Crude	201.1 ± 6.4	201.3 ± 5.7	223.7 ± 8.5	0.03	212.4 ± 5.8	198.5 ± 6.4	214.4 ± 8.2	0.21	215.6 ± 7.6	199.7 ± 5.7	209.9 ± 7.4	0.27
Model 1^a^	193.8 ± 7.6	198.0 ± 6.9	236.7 ± 8.0	0.001	212.3 ± 7.2	200.5 ± 7.0	215.2 ± 6.8	0.30	218.5 ± 7.8	200.6 ± 7.1	208.5 ± 7.9	0.23
Model 2^b^	202.0 ± 11.0	214.8 ± 9.9	212.8 ± 11.9	0.67	206.5 ± 10.2	213.8 ± 9.8	208.5 ± 10.2	0.87	215.4 ± 11.0	203.0 ± 9.9	211.2 ± 11.2	0.68
Model 3^c^	201.1 ± 11.3	214.9 ± 10.1	212.3 ± 12.1	0.64	206.6 ± 10.5	213.1 ± 10.1	208.3 ± 10.3	0.90	214.5 ± 11.2	203.1 ± 10.0	211.0 ± 11.3	0.73
Model 4^d^	199.9 ± 11.6	215.4 ± 10.3	213.0 ± 12.4	0.58	206.4 ± 10.9	213.6 ± 10.4	207.9 ± 10.7	0.88	215.2 ± 11.5	203.5 ± 10.3	210.0 ± 11.5	0.75
sVCAM-1 (mg/L)												
Crude	502.5 ± 14.2	490.0 ± 12.6	496.9 ± 14.0	0.81	505.7 ± 14.2	505.0 ± 15.1	480.2 ± 11.6	0.31	505.7 ± 14.2	494.8 ± 14.7	489.3 ± 12.0	0.68
Model 1^a^	496.8 ± 15.6	486.3 ± 14.2	504.2 ± 16.3	0.69	504.3 ± 14.5	506.9 ± 14.1	477.8 ± 13.7	0.26	502.1 ± 15.6	498.1 ± 14.3	487.2 ± 15.8	0.81
Model 2^b^	506.4 ± 30.4	483.0 ± 27.3	488.1 ± 32.9	0.84	477.4 ± 27.1	547.3 ± 26.1	448.1 ± 27.2	0.03	493.2 ± 30.2	487.5 ± 27.3	497.2 ± 30.8	0.97
Model 3^c^	501.6 ± 30.7	484.9 ± 27.4	483.8 ± 33.0	0.90	479.8 ± 27.7	541.2 ± 26.7	446.1 ± 27.2	0.05	484.9 ± 30.6	489.3 ± 27.3	496.1 ± 30.7	0.97
Model 4^d^	498.9 ± 31.4	486.3 ± 28.0	485.2 ± 33.7	0.94	475.8 ± 28.4	546.7 ± 27.1	444.2 ± 27.9	0.03	490.3 ± 31.2	487.0 ± 27.8	493.1 ± 31.2	0.99

All values are means ± SE. P were obtained from analysis of covariance (ANCOVA). *Q* quartile, *E-selectin* endothelial selectin, *sICAM-1* soluble intercellular adhesion molecule-1, *sVCAM-1* soluble vascular cell adhesion molecule-1.

^a^Model 1: Adjusted for age and energy intake.

^b^Model 2: Further adjusted for physical activity (MET- h/wk), current corticoid steroids use (yes or no), current OCP use (yes or no), marital status (categorical), menopausal status (yes or no), systolic blood pressure, diastolic blood pressure, and socioeconomic status (categorical).

^c^Model 3: Further adjusted for BMI.

^d^Model 4: Additionally adjusted for blood lipids (serum triglyceride, serum total cholesterol, HDL, and LDL-cholesterol) and glucose.

The linear associations of dietary nutrient patterns with insulin resistance and endothelial function indices are reported in Table 6. A significant increase in values of sICAM-1 was seen along with each one increase in tertiles of NP1, in the crude model (B = 11.16, 0.95% CI 1.45, 20.87). This association was also significant in model 1, after adjustment for age and energy intake (B = 21.61, 0.95% CI 9.76, 33.45). However, this association disappeared after further adjustment for other potential variables. There was no linear association between NP2 and markers of insulin resistance and endothelial function. Furthermore, each increase in tertiles of NP3 was associated with a marginal increase in HOMA-IR values in model 3 (B = 0.42, 0.95% CI 0.00, 0.84). This association was removed after adjustment for endothelial function markers in model 4 (B = 0.40, 95% CI − 0.02, 0.83).

**Table 6:** Linear association of nutrient dietary patterns^1^ with insulin resistance and endothelial function indexes.

	NP^1^		NP^2^		NP^3^	
	B (95% CI)	P	B (95% CI)	P	B (95% CI)	P
FBG (mg/dL)						
Crude	0.22 (− 1.36, 1.82)	0.77	1.32 (− 0.38, 2.82)	0.13	− 0.31 (− 1.91, 1.28)	0.70
Model 1^a^	0.44 (− 2.02, 2.11)	0.96	1.30 (− 0.35, 2.96)	0.12	− 0.65 (− 2.66, 1.35)	0.52
Model 2^b^	− 0.16 (− 4.42, 4.08)	0.93	− 0.03 (− 3.34, 3.26)	0.98	0.78 (− 3.15, 4.72)	0.69
Model 3^c^	0.52 (− 4.25, 4.35)	0.98	0.75 (− 3.27, 3.42)	0.96	0.94 (− 3.03, 4.91)	0.64
Model 4^d^	0.17 (− 4.34, 4.69)	0.93	− 0.05 (− 3.66, 3,55)	0.97	1.23 (− 3.00, 5,47)	0.56
Insulin (mU/L)						
Crude	− 0.62 (− 1.79, 0.54)	0.29	− 0.50 (− 1.67, 0.66)	0.39	− 0.59 (− 1.75, 0.57)	0.31
Model 1^a^	− 0.13 (− 1.62, 1.35)	0.85	− 0.52 (− 1.72, 0.67)	0.39	0.31 (− 1.14, 1,77)	0.66
Model 2^b^	− 1.75 (− 3.80, 0.28)	0.09	− 0.72 (− 2.34, 0.89)	0.37	1.74 (− 0.16, 3,65)	0.07
Model 3^c^	− 1.63 (− 3.70, 0.43)	0.12	− 0.67 (− 2.31, 0.97)	0.41	1.85 (− 0.06, 3.78)	0.05
Model 4^d^	− 1.59 (− 3.66, 0.48)	0.13	− 0.76 (− 2.42, 0.89)	0.36	1.72 (− 0.21, 3.66)	0.08
HOMA-IR						
Crude	− 0.11 (− 0.36, 0.14)	0.38	− 0.08 (− 0.34, 0.16)	0.50	− 0.13 (− 0.38, 0.11)	0.29
Model 1^a^	− 0.01 (− 0.33, 0.31)	0.94	− 0.08 (− 0.34, 0.17)	0.50	0.03 (− 0.28, 0.35)	0.81
Model 2^b^	− 0.37 (− 0.82, 0.08)	0.10	− 0.19 (− 0.55, 0.16)	0.27	0.39 (− 0.02, 0.81)	0.06
Model 3^c^	− 0.33 (− 0.79, 0.12)	0.14	− 0.18 (− 0.54, 0.18)	0.31	0.42 (0.00, 0.84)	0.05
Model 4^d^	− 0.32 (− 0.78, 0.13)	0.16	− 0.21 (− 0.57, 0.16)	0.26	0.40 (− 0.02, 0.83)	0.06
HOMA-β						
Crude	− 82.82 (− 166.95, 1.29)	0.05	− 62.41 (− 146.95, 22.13)	0.14	29.23 (− 54.93, 113.40)	0.49
Model 1^a^	− 91.60 (− 201.16, 17.94)	0.10	− 64.18 (− 152.90, 24.53)	0.15	86.58 (− 21.15, 194.31)	0.11
Model 2^b^	− 239.55 (− 534.39, 55.27)	0.11	− 19.72 (− 252.96, 213.52)	0.86	241.89 (− 33.01, 516.80)	0.08
Model 3^c^	− 228.93 (− 527.01, 69.15)	0.13	5.52 (− 231.75, 242.81)	0.96	237.31 (− 39.89, 514.53)	0.09
Model 4^d^	− 241.66 (− 538.15, 54.83)	0.10	− 22.32 (− 260.99, 216.34)	0.85	236.16 (− 41.80, 514.13)	0.09
QUICKY						
Crude	− 0.002 (− 0.008, 0.005)	0.62	− 0.005 (− 0.01, 0.002)	0.15	− 0.002 (− 0.009, 0.005)	0.54
Model 1^a^	− 0.001 (− 0.01, 0.007)	0.77	− 0.004 (− 0.01, 0.002)	0.20	− 0.003 (− 0.01, 0.005)	0.46
Model 2^b^	0.007 (− 0.009, 0.02)	0.38	− 0.005 (− 0.01, 0.008)	0.48	− 0.01 (− 0.02, 0.004)	0.14
Model 3^c^	0.006 (− 0.01, 0.02)	0.47	− 0.005 (− 0.01, 0.008)	0.41	− 0.01 (− 0.02, 0.003)	0.11
Model 4^d^	0.007 (− 0.01, 0.02)	0.43	− 0.004 (− 0.01, 0.009)	0.51	− 0.01 (− 0.02, 0.003)	0.10
E-selectin (mg/L)						
Crude	− 6.15 (− 13.18, 0.87)	0.08	− 9.35 (− 16.36, − 2.34)	0.009	0.51 (− 6.51, 7.54)	0.88
Model 1^a^	− 3.32 (− 11.80, 5.14)	0.44	− 7.49 (− 14.30, − 0.68)	0.03	3.11 (− 5.22, 11.45)	0.46
Model 2^b^	− 4.32 (− 15.20, 6.55)	0.43	0.03 (− 8.54, 8.61)	0.99	5.86 (− 4.28, 16.01)	0.25
Model 3^c^	− 3.71 (− 14.70, 7.28)	0.50	− 0.005 (− 8.72, 8.71)	0.99	6.60 (− 3.58, 16.79)	0.20
Model 4^d^	− 2.82 (− 14.06, 8.41)	0.61	− 1.31 (− 10.35,7.71)	0.77	5.71 (− 4.67, 16.10)	0.27
sICAM-1 (mg/L)						
Crude	11.16 (1.45, 20.87)	0.02	1.25 (− 8.57, 11.08)	0.80	− 2.73 (− 12.48, 7.02)	0.58
Model 1^a^	21.61 (9.76, 33.45)	˂ 0.001	1.34 (− 8.43, 11.12)	0.78	− 6.80 (− 18.67, 5.06)	0.26
Model 2^b^	6.03 (− 12.02, 24.08)	0.50	0.41 (− 13.80, 14.63)	0.95	− 1.69 (− 18.62, 15.23)	0.84
Model 3^c^	6.31 (− 12.03, 24.66)	0.49	0.04 (− 14.51, 14.60)	0.99	− 1.20 (− 18.35, 15.93)	0.88
Model 4^d^	7.33 (− 11.59, 26.25)	0.44	− 0.09 (− 15.33, 15.14)	0.99	− 2.28 (− 19.90, 15.33)	0.79
sVCAM-1 (mg/L)						
Crude	− 2.85 (− 21.89, 16.18)	0.76	− 12.90 (− 31.86, 6.04)	0.18	− 8.18 (− 27.11, 10.75)	0.39
Model 1^a^	2.48 (− 21.97, 26.94)	0.84	− 14.16 (− 33.75, 5.41)	0.15	− 6.80 (− 30.92, 17.30)	0.57
Model 2^b^	− 10.43 (− 60.26, 39.39)	0.67	− 12.97 (− 52.43, 26.48)	0.51	1.81 (− 45.14, 48.76)	0.93
Model 3^c^	− 9.66 (− 59.82, 40.49)	0.70	− 16.82 (− 56.87, 23.23)	0.40	5.58 (− 41.49, 52.66)	0.81
Model 4^d^	− 7.37 (− 58.86, 44.10)	0.77	− 15.76 (− 57.68, 26.16)	0.45	1.38 (− 46.70, 49.47)	0.95

All values are linear regression coefficient and 95% CIs, In tertiles, as continuous variables. P was obtained from linear regression analysis. *FBG* fasting blood glucose, *HOMA-IR* homeostatic model assessment of insulin resistance, *HOMA-β* homeostatic model assessment of beta-cell function, *QUICKI* quantitative insulin sensitivity check index, *E-selectin* endothelial selectin, *sICAM-1* soluble intercellular adhesion molecule-1, *sVCAM-1* soluble vascular cell adhesion molecule-1.

^a^Model 1: Adjusted for age and energy intake.

^b^Model 2: Further adjusted for physical activity (MET-h/wk), current corticoid steroids use (yes or no), current OCP use (yes or no), marital status (categorical), menopausal status (yes or no), systolic blood pressure, diastolic blood pressure, and socioeconomic status (categorical).

^c^Model 3: Further adjusted for BMI.

^d^Model 4: Glycemic variables (FBG, HOMA-IR, HOMA- β, QUICKI, and insulin) were further adjusted for adhesion molecules (E-selectin, sICAM-1, and sVCAM-1); serum adhesion molecules (E-selectin, sICAM-1, and sVCAM-1) were additionally adjusted for FBG, serum triglyceride, serum total cholesterol, HDL, and LDL-cholesterol.

Since no significant consistent association was observed between nutrient patterns and most of the indexes of both insulin resistance and endothelial dysfunction, the pathway analysis was not conducted in the current study.

## Discussion

In the current cross-sectional study, we illustrated that following two nutrient patterns was associated with insulin resistance and endothelial function indices. Such that, higher adherence to NP2, which consisted of chromium, selenium, copper, vitamin B6, MUFA, thiamin, vitamin D, and iron, considered as “legumes, nuts and protein foods nutrient pattern”, was associated with lower values of Insulin, HOMA-IR, and VCAM-1. Moreover, higher adherence to NP3 consisting of SFA, cholesterol, vitamin E, sodium, vitamin B12, zinc, and protein, named as “animal fat and meat + vitamin E nutrient pattern”, was associated with higher values of HOMA-β. Although HOMA-β is considered as an index of beta-cell function, its increased levels have shown to be associated with impaired glucose tolerance, type 2 diabetes, and insulin resistance^38,39^. Adhering to the third nutrient pattern in the current investigation has led to higher values of HOMA-β, but resulted in a reduction in QUICKY levels, a definite indicator of insulin resistance^40,41^, although this association was not statistically significant (P = 0.19). In addition, no linear association has been observed between tertiles of nutrient patterns and levels of glycemic and endothelial indices after considering all potential variables.

Obesity is known as an important risk factor for insulin resistance and prevalent around the world^42^. It has been declared that during recent years, a significant rise in prevalence of type 2 diabetes was concerning in some countries, despite lower numbers of obesity^42,43^. On the other hand, metabolic disorders such as hypertension and abdominal obesity^44,45^ are drastically associated with increased endothelial dysfunction and consequently coronary artery diseases^46^. So, it can be very important to find an effective way for managing these conditions. According to our study, following a diet rich in unsaturated fatty acids, copper, selenium, manganese, chromium, zinc, vitamin B6, thiamin, vitamin D, and dietary fiber, along with lower consumption of SFA, cholesterol, vitamin E, sodium, potassium, and vitamin B12 might help reduce risks of insulin resistance and endothelial dysfunction. More clinical trials are necessary to confirm these observations.

Previous studies have estimated the association between various nutrients and IR markers. For example, a prospective cohort study on 995 subjects has suggested a reduction in IR and hyperinsulinemia by following a nutrient pattern rich in potassium, vitamins B6, C, and A^24^. Moreover, significant inverse associations were observed between adherence to the nutrient pattern rich in vitamin B and dietary fiber, and another pattern, called zinc, thiamin, and plant proteins with the values of glycated hemoglobin and fasting glucose in a prospective cohort study in South Africa^47^. Furthermore, another observational study among Iranian overweight and obese adolescents has reported an increased risk of metabolically unhealthy obesity as well as an increment in HOMA-IR levels through following a “high fat and sodium” nutrient pattern^48^. Moreover, it has been reported that following a diet with a higher Mediterranean-style score, rich in MUFA, PUFA, nuts, and seeds in children, might be associated with lower levels of HOMA-IR, fat mass index (FMI), and cardiometabolic risk in their adulthood^49^. Another 3-year prospective cohort study has found an inverse association between higher dietary approaches to stop hypertension (DASH) score and IR. DASH score was defined by higher intakes of legumes, nuts, fruits, and vegetables, and lower intakes of sodium, red and processed meat, and sweetened beverages in the mentioned study^50^. A meta-analysis of 44 trial and prospective cohort studies on patients with diabetes has also demonstrated a reduction in HbA1C and HOMA-IR levels in higher intakes of dietary fiber^51^. These investigations might confirm the favorable effect of NP2 in the current study (named legumes, nuts, and protein food) on levels of serum insulin and HOMA-IR. On the other hand, saturated fatty acids have been proven to increase the risk of insulin resistance^52^. Higher meat consumption was associated with an increase in HOMA and insulin levels in a population of non-diabetic women^53^. It has also been claimed that diets rich in animal protein might increase insulin resistance regardless of weight^54^. So, the increments in levels of HOMA-β in the present study across tertiles of NP3 (described as the meat and animal fat pattern) could be supported by these evaluations.

Several mechanisms might explain the association of nutrients with insulin resistance and endothelial dysfunction. Interventional studies have suggested that supplementation of zinc, selenium, and chromium might improve insulin resistance by reducing oxidative stress which can impair insulin secretion from β cells^55,56^. Additionally, it has been suggested that chromium might be able to increase insulin binding through increasing the number of insulin receptors and their phosphorylation^57^. The protective role of selenium against insulin resistance and type 2 diabetes might be associated with its ability to enhance the activity of glutathione peroxidase (GPx), which defends against reactive oxygen species (ROS)^58^. A combination of vitamin D3 and chromium has also shown to decrease HOMA-IR levels by regulation of inflammatory markers like TNF-α^16^. On the other hand, MUFA consumption has a favorable effect on sVCAM-1 through the reduction in NF-kB, another marker of oxidative stress^59,60^. Co-supplementation of omega 3 fatty acids and chromium could also enhance endothelial function by preventing the activity of phospholipase A2, a prooxidant enzyme, and provoking antioxidant enzymes^61^. A randomized control trial on 124 children with type 1 diabetes documented that folate and vitamin B6 supplementation had a positive effect on endothelial function, because folate supplementation could enhance levels of tetrahydrobiopterin, a substantial cofactor for NO synthesis^62^. Furthermore, vitamin B6 could regulate the inflammatory response^63^. Vitamin D and its receptors (VDRs) could also enhance endothelial function by increasing NO synthesis, through a positive regulation in the activity of endothelial Nitric Oxide Synthase (eNOS)^64^.

As far as we know, this is the first study investigating the association of various NPs with insulin resistance and endothelial dysfunction. Moreover, validated questionnaires were used to assess dietary intakes and covariates. Nevertheless, some limitations can be acknowledged in our study. Considering cross-sectional design of the study, causal relationships could not be confirmed. Since the current investigation was conducted on a population of nurses living in Isfahan, generalizing the results to all Iranian women might not be totally possible. Although was controlled for several confounders in the analyses, the effect of residual confounders might not be avoided. In addition, misclassification and measurement errors are unavoidable due to the self-reported design of questionnaires. Finally, the study was carried out on a particular group of people (female nurses working in hospitals) and its findings could not be generalized to the whole adult population.

In conclusion, in the current cross-sectional study higher adherence to the second nutrient pattern, associated with higher intakes of chromium, selenium, copper, vitamin B6, MUFA, PUFA, vitamin D, and iron was associated with lower Insulin, HOMA-IR, and VCAM-1 values. However, higher adherence to the third nutrient pattern, rich in SFA, cholesterol, vitamin E, sodium, and vitamin B12 was associated with higher HOMA-β values. Considering the findings of the current study, adhering to a nutrient dietary pattern, rich in selenium, copper, iron, vitamin B6, vitamin D, and unsaturated fatty acids (including PUFAs and MUFAs) with lower intakes of cholesterol, sodium, vitamins E and B12, and saturated fatty acids can reduce the risk of insulin resistance and endothelial dysfunction in female population. However, further prospective investigations are required to affirm these associations.

## Data Availability

The data that support the findings of this study are available from the corresponding authors upon reasonable request.

## Abbreviations

NO: Nitric oxide
IR: Insulin resistance
USDA: US Department of Agriculture database
sICAM-1: Soluble intercellular adhesion molecule 1
sVCAM-1: Soluble vascular adhesion molecule 1
ELISA: Enzyme-linked immunosorbent assay
FBG: Fasting blood glucose
BMI: Body Mass Index
IPAQ: International Physical Activity Questionnaire
KMO: Kaiser–Meyer–Olkin
SBP: Systolic blood pressure
DBP: Diastolic blood pressure
FFQ: Food frequency questionnaire
HOMA-β: Homeostasis model assessment-β
HOMA-IR: Homeostasis model assessment-Insulin resistance
MetS: Metabolic syndrome
ANCOVA: Analysis of covariance
SPSS: Statistical package for the social sciences
SD: Standard deviation
SE: Standard error
95% CI: 95% confidence interval
MUFA: Monounsaturated fatty acids
PUFA: Polyunsaturated fatty acids
SFA: Saturated fatty acids
WHO: World Health Organization
WC: Waist circumference
BP: Blood pressure
TG: Triglycerides
HDL-c: High-density lipoprotein cholesterol
ANOVA: Analysis of variance
